# Scalable Purification and Characterization of the Anticancer Lunasin Peptide from Soybean

**DOI:** 10.1371/journal.pone.0035409

**Published:** 2012-04-13

**Authors:** Lauren E. Seber, Brian W. Barnett, Elizabeth J. McConnell, Steven D. Hume, Jian Cai, Kati Boles, Keith R. Davis

**Affiliations:** 1 James Graham Brown Cancer Center, University of Louisville, Louisville, Kentucky, United States of America; 2 Owensboro Cancer Research Program, Owensboro, Kentucky, United States of America; 3 Kentucky BioProcessing, LLC, Owensboro, Kentucky, United States of America; 4 Department of Pharmacology and Toxicology, University of Louisville, Louisville, Kentucky, United States of America; University of Helsinki, Finland

## Abstract

Lunasin is a peptide derived from the soybean 2S albumin seed protein that has both anticancer and anti-inflammatory activities. Large-scale animal studies and human clinical trials to determine the efficacy of lunasin *in vivo* have been hampered by the cost of synthetic lunasin and the lack of a method for obtaining gram quantities of highly purified lunasin from plant sources. The goal of this study was to develop a large-scale method to generate highly purified lunasin from defatted soy flour. A scalable method was developed that utilizes the sequential application of anion-exchange chromatography, ultrafiltration, and reversed-phase chromatography. This method generates lunasin preparations of >99% purity with a yield of 442 mg/kg defatted soy flour. Mass spectrometry of the purified lunasin revealed that the peptide is 44 amino acids in length and represents the original published sequence of lunasin with an additional C-terminal asparagine residue. Histone-binding assays demonstrated that the biological activity of the purified lunasin was similar to that of synthetic lunasin. This study provides a robust method for purifying commercial-scale quantities of biologically-active lunasin and clearly identifies the predominant form of lunasin in soy flour. This method will greatly facilitate the development of lunasin as a potential nutraceutical or therapeutic anticancer agent.

## Introduction

Lunasin has been described as a 43 amino-acid peptide that is encoded within the soybean GM2S-1 gene and was first identified as a novel peptide found in soybean seed extracts [1]. Initial studies of the biological activity of lunasin found that expression constructs encoding the lunasin peptide sequence resulted in arrested cell division and the formation of nonseptated filaments in *E. coli* and caused mitotic arrest in mammalian cell lines, apparently by binding to kinetochore regions of the centromere and blocking microtubule attachment [2]. These initial results suggested that lunasin could be useful as a cancer therapeutic provided that lunasin could be specifically delivered to cancer cells. Given that consumption of soy products has been associated with the reduced incidence of specific cancers [3], [4]; additional studies were done to examine the cancer chemoprevention activity of lunasin. In a series of key studies, addition of a synthetic lunasin peptide to mammalian cells prevented cellular transformation by chemical carcinogens and the viral oncogenes *ras* and E1A [5], [6], [7], [8]. An interesting observation made during these initial studies was that neither normal immortalized cells, nor stable cancer cell lines were affected by lunasin peptide exposure. These results provided the initial indication that lunasin may be used as a chemoprevention agent. This hypothesis was further supported by animal studies in which topical application of lunasin significantly suppressed skin papilloma formation in SENCAR (SENsitivity to CARcinogenesis) mice treated with a combination of the chemical carcinogen 7,12-dimethylbenz[α]anthracene and the tumor promoter 12-O-tetradecanoylphorbol-13-acetate [5]. Since the original discovery of lunasin in soybean, lunasin has been identified in barley, wheat, *Solanum nigrum*, and amaranth [7], [9], [10], [11], [12], [13]. Analysis of different soybean cultivars demonstrated that lunasin content varied significantly, suggesting that it may be possible to breed soybean varieties with higher lunasin content [14], [15].

More recent studies have demonstrated that lunasin can inhibit the growth of some cancer cells in culture and in a mouse xenograft model [16], [17], [18], [19] and that it also has anti-inflammatory activity [20], [21], [22]. This contradicts the earlier studies which were done on a limited number of cell lines and demonstrate that the initial conclusion that lunasin did not affect established cancer cells was incorrect. These latter studies suggest that lunasin may be useful both as a chemoprevention agent and a cancer therapeutic. Lunasin has been shown to bind specifically to the deacetylated core histones H3 and H4 and current hypotheses on lunasin's mechanism of action suggest that this is critical for the anticancer effects of lunasin [5], [7], [9], [23], [24], [25]. de Lumen and coworkers [6], [24], [26] have proposed a model for the molecular basis of the biological effects of lunasin based on the disruption of normal histone acetylation by histone deacetylase and histone acetylase. Recent studies have shown that treatment of cancer cells with lunasin may induce apoptosis through the intrinsic pathway [16], [17], [27] and that both the anti-inflammatory and anticancer effects are mediated by suppression of the NF-κB pathway [20], [28]. It is not known if these effects are linked to inhibition of HAT and disruption of histone acetylation. Recent gene expression studies indicate that lunasin can affect a number of signaling pathways in different cell types, thus, some of the observed biological effects of lunasin may be independent of histone acetylation [23], [29].

Although the potential anticancer effect of lunasin has been known for over a decade, little progress has been made to test *in vivo* efficacy of purified lunasin in animal or human clinical studies. One major limitation has been the lack of availability of the gram-kilogram quantities of highly purified lunasin required to conduct such studies. To address this need, we have developed a method for purifying lunasin from defatted soybean flour (white flake) that yields highly purified lunasin and can be easily scaled to produce kilogram quantities of peptide. The purified lunasin was biologically active as measured by histone binding assays and was found to have the same, if not higher, activity compared to synthetic lunasin. Structural analysis of the purified peptide revealed that the major form of lunasin present in soybean white flake is 44 amino acids in length and contains an additional C-terminal asparagine relative to previously published descriptions of lunasin.

## Results

### Establishment of extraction conditions

Previous reports describing the partial purification of lunasin utilized extraction of soy flour with water and phosphate buffered saline (PBS) [15], [30], [31]; however, a systematic analysis of extraction conditions was not described. We therefore tested the extraction efficiency of water and buffers using various extraction times, pH levels, and ratios of extraction solution volume to amount of white flake. These studies demonstrated that lunasin is readily extracted by both water and buffer solutions over a range of extraction conditions (Figure S1). Water and buffer solutions were found to have very similar extraction efficiencies and an extraction time as short as 30 minutes gave maximum yield of lunasin. Varying the ratio of extraction solution volume to amount of white flake over a range of 5∶1 to 12.5∶1 (buffer∶white flake) also did not have a significant effect on the amount of lunasin recovered. However, the lower buffer to white flake ratios gave more viscous extracts that were more difficult to work with. The only significant parameter observed was pH; lower pH buffers extracted slightly lower amounts of lunasin. Based on these results, and the fact that the subsequent anion-exchange chromatography step requires the sample to be in PBS, our standard extraction method utilized a modified PBS buffer (pH 7.4) at a 12.5∶1 buffer to white flake ratio with an extraction time of sixty minutes.

### Development of lunasin purification method

**Figure 1:**
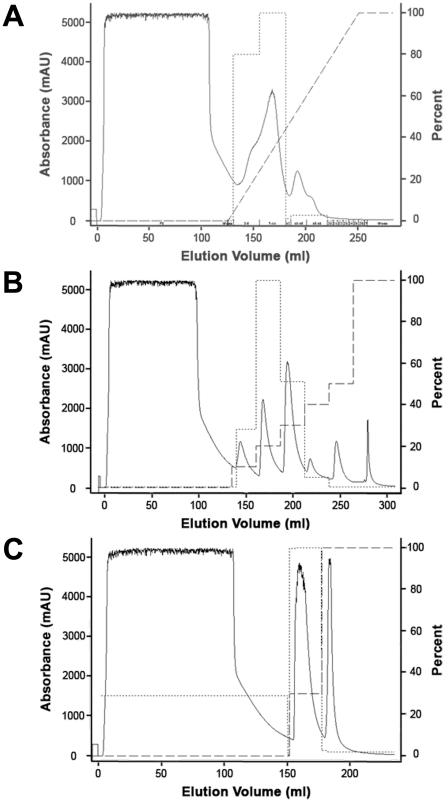
Optimization of anion-exchange chromatography method. (A) Elution of lunasin using a linear NaCl gradient. White flake was mixed with extraction buffer (75.5 mM sodium phosphate, 68.4 mM NaCl, 10 mM sodium metabisulfite, 20 mM ascorbic acid, pH 7.4) at a 12.5∶1 buffer to biomass ratio and mixed for one hour at 4°C. The mixture was filtered through four layers of cheesecloth and one layer of miracloth and then centrifuged at 10,000× *g* for 10 minutes at 4°C. The supernatant was collected, filtered through a 0.2 µm filter, and the clarified extract stored at 4°C until used. For chromatography, a 5 mL Q-Fast Flow HiTrap pre-packed anion-exchange column was equilibrated with ten CV of Buffer A (75.5 mM Sodium Phosphate, 68.4 mM NaCl, pH 7.4) prior to loading 100 mL of soy clarified extract. The column was washed with five CV of Buffer A to remove unbound proteins. A 25 CV linear salt gradient beginning at 68.4 mM and ending at 1000 mM NaCl at a flow rate of 5 mL/min was used to elute proteins from the column. Five mL fractions were collected during the elution. Fractions were pooled based on the A_280_ profile and analyzed for lunasin by ELISA and SDS-PAGE. The largest amount of lunasin was detected in fractions 7–11 which corresponds to NaCl concentrations of between 290 mM and 480 mM. (B) Development of a step-gradient elution method. Anion-exchange chromatography was performed using the same 5 mL Q-Fast Flow HiTrap column used in (A). After the column was stripped and re-equilibrated in Buffer A, 100 mL of clarified extract was applied, followed by eight CV washes with Buffer A. A step gradient consisting of six steps (10, 20, 30, 40, 50, and 100% Buffer B) of five CV each at of flow rate of 5 mL/min was used to elute lunasin. Each step fraction was analyzed for the presence of lunasin by ELISA and SDS-PAGE. Lunasin was detected in both the 20% and 30% B elution fractions which correspond to NaCl concentrations of between 255 mM and 348 mM. (C) Optimized step-gradient purification of lunasin using anion-exchange chromatography. A final step gradient chromatography run was performed as described in (B), except that a ten CV wash was done prior to the step elution. Two elution steps of 30% (5 CV) and 100% Buffer B (10 CV) which corresponded to NaCl concentrations of 348 mM and 1000 mM, respectively, were done. The presence of lunasin in each sample was determined by ELISA and SDS-PAGE. Lunasin was detected only within the 30% B elution fraction. Chromatograms show the A_280_ (solid line ^______^), percent Buffer B (^_ _ _ _ _^), and the percent maximum lunasin content as determined by ELISA (-------).

**Figure 2:**
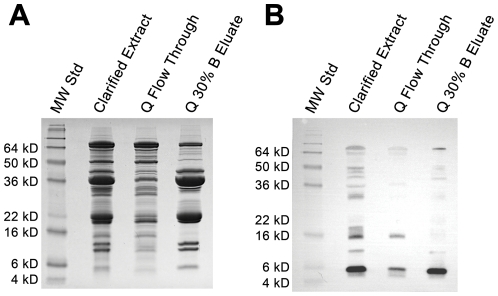
SDS-PAGE and immunoblot analysis of anion-exchanged purified lunasin. Aliquots of samples corresponding to the bench-scale anion-exchange chromatography method where lunasin was eluted using a step gradient (Figure 1C) were subjected to SDS-PAGE and immunoblot analysis. (A) SDS-PAGE of the clarified extract, column flow through (Q flow through), and the 30% Buffer B elution (Q 30% B Fraction). Clarified extract, Q flow through, and Q-30%B fraction were prepared at dilutions of 1∶8, 1∶8, and 1∶10, respectively, and electrophoresed using 15% Tris-glycine gels. Molecular weight standards (MW Std) are shown in the first lane. (B) Immunoblot analysis of the clarified extract, Q flow through, and the Q 30% B Fraction. Proteins were separated by SDS-PAGE as described in (A), transferred to a PVDF membrane, and probed with a lunasin-specific mouse monoclonal antibody. For SDS-PAGE, clarified extract, Q flow through, and Q-30% B fraction were prepared at dilutions of 1∶20, 1∶20, and 1∶40, respectively. Molecular weight standards (MW Std) are shown in the first lane.

Previously published results [30], [31] and our own preliminary studies indicated that anion-exchange chromatography was an effective method for obtaining partially purified lunasin. Thus, we optimized conditions for fractionation of lunasin using Q-Sepharose FF chromatography. Initial experiments where lunasin was eluted from the Q-Sepharose FF column using a linear gradient of NaCl (Figure 1A) demonstrated that lunasin eluted between 0.29 and 0.48 M NaCl. To simplify the large-scale purification, we utilized these results to develop a step-elution method for fractionating lunasin by Q-Sepharose FF chromatography (Fig. 1B). This study demonstrated that a step elution using 0.35 M NaCl effectively eluted lunasin from the column and yielded a partially purified preparation enriched for lunasin (Fig. 1C, Fig. 2A and 2B).

**Figure 3:**
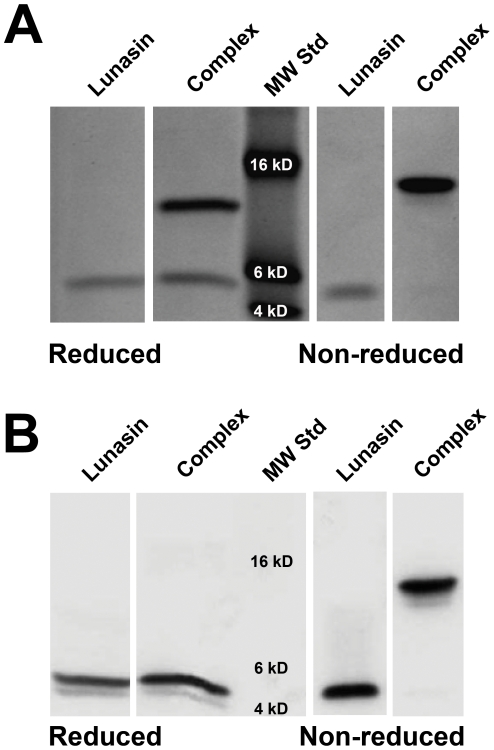
Detection of lunasin within a 14 kDa protein complex using SDS-PAGE and immunoblot analyses. Coomassie-stained SDS-PAGE gel (A), and corresponding immunoblot (B) of purified lunasin-containing complex under reducing and non-reducing conditions. The first two lanes represent lunasin (5.1 kDa) and lunasin-containing complex (14.1 kDa) under standard reducing conditions while the 4^th^ and 5th lanes represent equivalent samples under non-reducing conditions (without BME in the sample buffer). Lanes with lunasin contain 300 ng of synthetic lunasin as a reference, while lanes with complex contain 3 µg of lunasin-containing complex. Identification of the lunasin-containing complex by immunoblot analysis was accomplished using a 1∶5000 dilution of rabbit polyclonal anti-lunasin as the primary antibody and a 1∶100,000 dilution of HRP-conjugated goat anti-rabbit as the secondary antibody. Molecular weight standards (MW Std) are shown in the 3rd lane.

**Figure 4:**
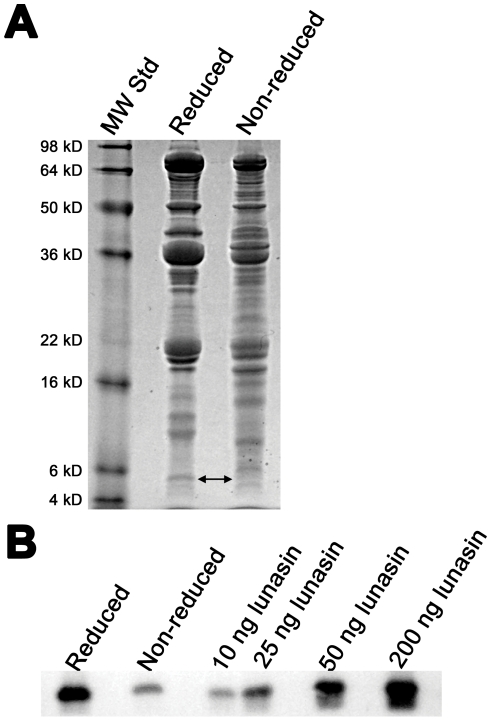
Quantitation of lunasin and lunasin complex in clarified extracts. White flake (120 g) was extracted with 1.5 L of 75.5 mM sodium phosphate/150 mM NaCl/20 mM ascorbic acid/10 mM sodium metabisulfite, pH 7.4 for one hour. A clarified extract was produced by treating the initial extract with Celpure P100 and filtration using one micron M-503 filter pads. (A) SDS-PAGE analysis of reduced and non-reduced clarified extract. The clarified extract was diluted 1∶10 and analyzed by SDS-PAGE using a 15% Tris-glycine gel under standard reducing and non-reducing (without BME in the sample buffer) conditions. Molecular weight standards (MW Std) are shown in the first lane. The arrow indicates a ∼5 kDa band that corresponds to lunasin. The lack of a clear lunasin band in the sample analyzed under non-reducing conditions indicates that most of the lunasin present in the clarified extract is in protein complexes stabilized by disulfide bridges. (B) Immunoblot analysis of reduced and non-reduced clarified extract. Clarified extract was diluted 1∶10 and subjected to SDS-PAGE along with a series of synthetic lunasin standards as described in (A). The separated proteins were transferred to a PVDF membrane and probed with a lunasin-specific mouse monoclonal antibody diluted 1∶100,000. The amount of lunasin present was determined by image analysis using the synthetic lunasin band intensities to generate a standard curve. This analysis demonstrated that <20% of the extractable lunasin is present in the ∼5 kDa form.

During our analysis of the Q-Sepharose FF purified lunasin fractions by sodium dodecyl sulfate polyacrylamide gel electrophoresis (SDS-PAGE), we observed that the ∼5 kDa lunasin peptide always co-purified with a ∼9 kDa protein. Analysis of the Q-Sepharose FF purified lunasin by SDS-PAGE under standard reducing conditions or in the absence of beta-mercaptoethanol (BME) in conjunction with immunoblotting revealed that the majority of lunasin present in the partially purified preparation was in a ∼14 kDa protein complex (Figure 3). Analyses of clarified extracts confirmed that approximately 80% of the extractable lunasin is in this ∼14 kDa complex (Figure 4). This critical observation revealed that to obtain the maximum amount of lunasin from white flake, and presumably other plant-derived sources, it is necessary to include a reducing step to release lunasin from the ∼14 kDa complex.

To test the ability of various reducing agents to disrupt the lunasin-containing complex, we generated a highly purified lunasin-containing complex using Q-Sepharose FF chromatography followed by ultrafiltration using a 50 kDa molecular weight cut-off (MWCO) membrane. The purified complex was treated with varying concentrations of BME, tris(2-carboxyethyl)phosphine (TCEP) and dithiothreitol (DTT), then analyzing the resulting protein profiles by SDS-PAGE (Figure S2). All three reducing agents were effective in releasing lunasin from the ∼14 kDa complex. Based on its suitability for use in the large-scale production of lunasin, 2 mM DTT was selected to reduce the Q-Sepharose FF purified lunasin prior to further purification.

**Figure 5:**
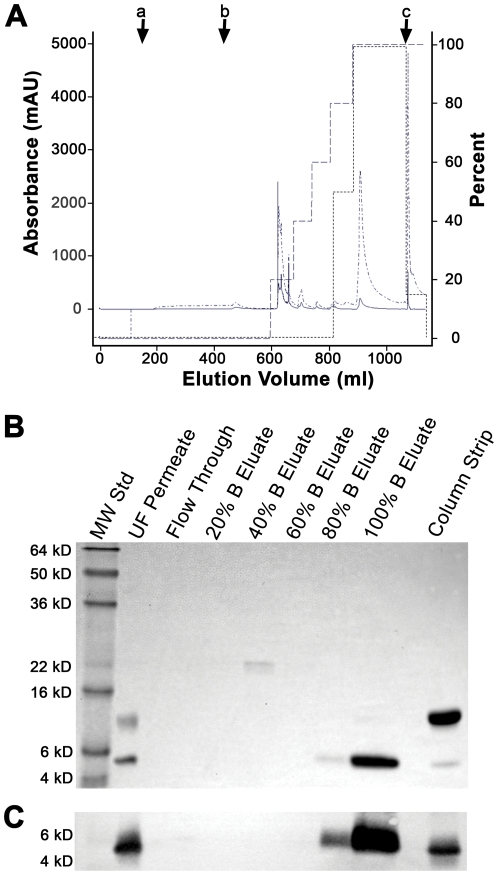
Reversed-phase chromatography (RPC) method development. Bench-scale RPC was performed using a 1.6×8.0 cm Source15RPC column that was sanitized with 1 N NaOH and equilibrated with ten CV of Buffer A (75.5 mM sodium phosphate/68.4 mM NaCl, pH 7.4) prior to sample load. The ultrafiltration (UF) permeate was brought to a final concentration of 1 M ammonium sulfate and then applied to the column followed by a five CV wash with Buffer A. Bound proteins were eluted using a five-step gradient consisting of 20%, 40%, 60%, 80%, and 100% Buffer B (64.2 mM sodium phosphate/58 mM NaCl/15% n-propanol (v/v)). Each gradient step was approximately five CV except the 100% B step which was ten CV. The column was then stripped using 65% n-propanol. (A) RPC of the UF permeate. Letters with arrows represent beginning of (a) sample load, (b) column wash, and (c) column strip. The presence of lunasin in each sample was determined by ELISA and SDS-PAGE. Lunasin was detected primarily within the 100% B elution fraction. Chromatogram shows the A_280_ (solid line ^______^), the A_215_ (^.__.^^__.^), percent Buffer B (^_ _ _ _ _^), and the percent maximum lunasin content as determined by ELISA (-------). (B) Coomassie-stained SDS-PAGE gel of RPC fractions. SDS-PAGE using a 15% Tris-glycine gel was performed on 1∶10 dilution and 1∶4 dilutions of the UF permeate and column strip, respectively, and undiluted samples from the column flow through and Buffer B step gradient fractions. Molecular weight standards (MW Std) are shown in the first lane. The majority of lunasin (>95%) was detected in the 100% B eluate, with minor amounts detected in the 80% B eluate and in the column strip. The major contaminating ∼9 kDa protein was detected exclusively in the column strip. (C) Immunoblot analysis of the UF permeate, column flow through, step gradient fractions, and column strip. Proteins separated by SDS-PAGE were transferred to a PVDF membrane and probed with a lunasin-specific mouse monoclonal antibody. For SDS-PAGE, dilutions of the UF permeate (1∶10), 80% B eluate (1∶4), 100% B eluate (1∶4), and column strip (1∶4) were made. All other samples were undiluted. The position of the 4 kDa and 6 kDa molecular weight standards (MW Std) are shown in the first lane.

Since many of the contaminating proteins still present in the Q-Sepharose FF purified lunasin preparation were >20 kDa, ultrafiltration using a 30 kDa MWCO membrane was performed. In this application of ultrafiltration, lunasin accumulates in the permeate. This ultrafiltration step removed most of the contaminating proteins except for the ∼9 kDa protein that is a component of the ∼14 kDa lunasin-containing complex. To further purify lunasin, we tested the ability of reversed-phase chromatography to remove the remaining contaminating proteins (Figure 5A). This polishing step was found to be very effective and resulted in a lunasin preparation that was >99% pure as assessed by analysis of Coomassie-stained SDS-PAGE gels (Figure 5B). Immunoblot analysis using a lunasin-specific antibody provided an initial confirmation that the purified protein was indeed lunasin (Figure 5C).

### Mass spectrometry of purified lunasin and the lunasin complex

**Figure 6:**
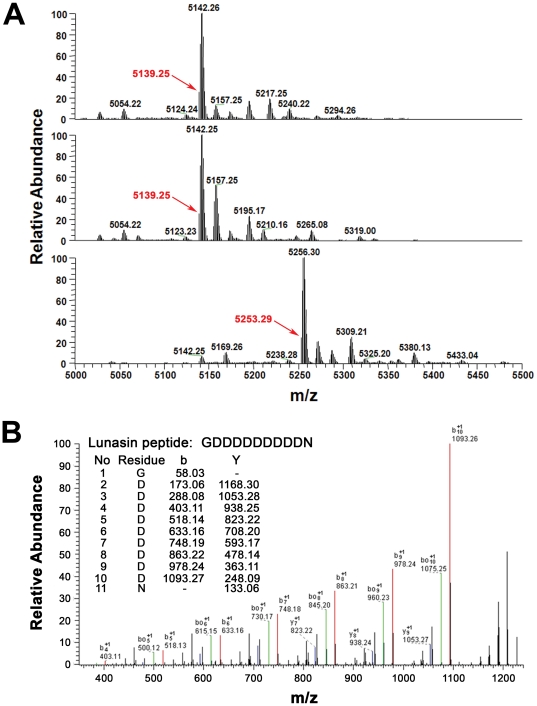
Mass spectrometry of the purified lunasin. (A, top panel) Deconvoluted MS Spectra of purified lunasin. The monoisotopic mass of the purified lunasin was found to be 5139.25 Da, which is 114.02 Da higher than the expected monoisotopic mass (5025.23 Da) for the 43 amino-acid form of lunasin described in the literature. The mass difference suggests that the predominant form of our purified lunasin contains 44 amino acids and that it contains an additional asparagine residue. (A, middle panel) Deconvoluted spectrum of lunasin reduced with DTT. Reduction with DTT did not cause a mass shift, indicating there is no disulfide bond present in the purified lunasin. (A, bottom panel) Deconvoluted spectrum of lunasin complex treated with DTT and IAA. The monoisotopic mass of lunasin shifted to 5253.29 Da after alkylation with IAA, which is 114.04 Da higher than unalkylated lunasin. This mass shift confirmed that lunasin has two free cysteine residues as expected. (B) MS/MS spectrum of C-terminal peptide of lunasin. Calculated b and Y ions for the peptide GDDDDDDDDDN are shown in the table inset. The matched b (red) and Y (blue) ions detected match very well the expected fragment ion values for this peptide. Signals corresponding to the loss of one (green) or more H_2_O molecules, which are expected in MS/MS spectra of peptides with multiple acidic residues, are also evident in the spectrum. These [b – H_2_O] signals are consistent with the presence of the GDDDDDDDDDN peptide. This analysis confirmed that the residue at the C-terminus of lunasin purified from soybean is asparagine rather than aspartic acid.

To confirm the identity of our purified ∼5 kDa protein as lunasin and to attempt to determine the identity of the ∼9 kDa protein that is present in the lunasin complex, we performed electrospray ionization mass spectrometry (ESI-MS) on the purified lunasin and the lunasin complex. The monoisotopic mass of the most abundant peptide in the purified lunasin sample was found to be 5139.25 Da, which is 114.02 Da higher than the expected monoisotopic mass of 5025.23 Da for the 43 amino-acid form of lunasin described in the literature (Figure 6A). The mass difference suggests that the predominant form of our purified lunasin contains 44 amino acids and that it contains an additional asparagine residue. A very weak signal was observed with a monoisotopic mass 5025.23 Da suggesting that a minor amount of the 43 amino-acid form of lunasin may be present. ESI-MS analysis of the purified lunasin after treatment with DTT or DTT in combination with iodoacetamide (IAA) gave monoisotopic masses of 5139.25 Da and 5253.29 Da, respectively, and indicate that the purified lunasin does not contain any disulfide bonds and contains two cysteine residues as expected. To further confirm that the purified lunasin is a 44 amino acid peptide, we digested the purified lunasin with trypsin and acquired LC-MS/MS spectra of the resulting tryptic peptides. Peptides identified in this analysis are summarized in Table 1. The identified peptides covered 75% of the purified lunasin and included a peptide with an *m/z* of 1225.32, which is consistent with the sequence GDDDDDDDDDN. A peptide with an *m/z* of 1111.28 corresponding to the sequence GDDDDDDDDD was not detected. Analysis of the MS/MS spectrum of the *m/z* 1225.32 peptide by (Figure 6B) confirmed that this peptide has the sequence GDDDDDDDDDN and thus, that the predominant form of lunasin isolated from white flake has the sequence SKWQHQQDSCRKQLQGVNLTPCEKHIMEKIQGRGDDDDDDDDDN.

**Table 1:** Peptides identified from lunasin complex subunits by LC-MS/MS analysis.

Subunit	Peptide	MH+	ΔM (ppm)	z	P
**5.14 kDa**	QLQGVNLTPCEK	1386.7046	−6.12	2	9.67E-09
	QLQGVNLTPCEK	1386.7046	−7.07	3	6.97E-04
	KQLQGVNLTPCEK	1514.7995	−7.45	2	2.73E-08
	KQLQGVNLTPCEK	1514.7995	−5.42	3	1.63E-03
	WQHQQDSCR	1244.5225	−7.37	2	4.71E-08
	GDDDDDDDDDN	1225.3247	−0.01	1	5.15E-03
**8.98 kDa**	ELINLATMCR	1220.6126	−6.17	2	6.36E-08
	ELINLATM*CR	1236.6075	−6.54	2	1.31E-07
	IMENQSEELEEK	1478.6679	−4.47	2	1.85E-07
	IM*ENQSEELEEK	1494.6628	−4.29	2	1.25E-05
	CCTEMSELR	1185.4697	−5.80	2	4.21E-07
	CCTEM*SELR	1201.4646	−6.67	2	4.70E-05
	FGPMIQCDLSSDD	1484.6032	−6.26	2	2.10E-06
	FGPM*IQCDLSSDD	1500.5981	−5.53	2	5.27E-06

M* indicates oxidized methionine; MH^+^ is the monoisotopic mass calculated from peptide sequence; ΔM is the error of detected MH^+^ in ppm; z is the charge state of the ion from which MS/MS spectrum was generated; and P is the probability that MS/MS spectrum matched the sequence randomly. Note that alkylation of cysteine by IAA increases the mass of cysteine by 57 Da.

**Figure 7:**
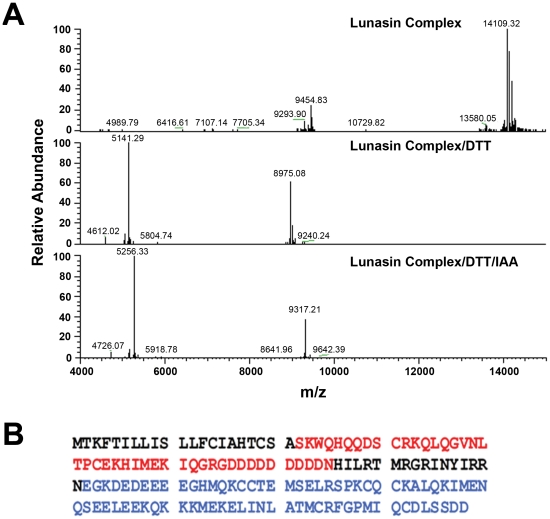
Mass spectrometry of the purified lunasin-containing complex. (A, top panel) Deconvoluted spectrum of purified lunasin complex. The most abundant isotopic mass in the spectrum is at 14109.3 Da. The mass signal adjacent to lunasin complex (14207.3 Da) is the adduct of lunasin complex with phosphoric acid (plus 98 Da). (A, middle panel) Deconvoluted spectrum of reduced lunasin complex. The most abundant isotopic masses shown in the spectrum are lunasin (5141.3 Da) and soybean albumin long chain (8975.1 Da). (A, bottom panel) Deconvoluted spectrum of lunasin complex treated with DTT and IAA. The most abundant masses shown in the spectrum are lunasin (5256.3 Da) and soybean albumin long chain (9317.2 Da). The monoisotopic masses are 5139.28 Da and 5253.33 Da for lunasin and lunasin treated with DTT and IAA respectively. The monoisotopic masses of lunasin complex and soybean albumin long chain were too low to be detected. (B) Sequence of 2S albumin preproprotein. Sequence in red is corresponds to our purified lunasin and its monoisotopic and average molecular weights are 5139.27 and 5142.43 Da, respectively. Sequence in blue corresponds to soybean albumin long chain and its monoisotopic and average molecular weights are 8969.05 and 8975.17 Da, respectively.

ESI-MS analysis of the purified ∼14 kDa lunasin complex revealed that the most abundant isotopic mass of the complex was 14109.3 Da (Figure 7A). Upon reduction of the complex with DTT, the most abundant isotopic masses corresponding to the two subunits of the complex were 5141.3 Da and 8975.1 Da Figure 7A). The sum of the average molecular weights of the subunits, 14116.4 Da, is 7 Da higher than lunasin complex (14109.3 Da), indicating that there are four disulfide bonds in lunasin complex. Although reduction of the four disulfide bonds should have caused an 8 Da mass shift, the observed shift of 7 Da is likely due to the change of isotope mass profiles with the size of the peptides. A more precise method for calculating the mass shift due to reduction would be to use the monoisotopic masses. However, the signals for the monoisotopic masses of the lunasin complex and 8975.1 Da subunit were too weak to be detected. ESI-MS analysis of the lunasin complex after reduction with DTT and alkylation with IAA demonstrated that the 5141.3 Da and 8975.1 Da complex subunits contain two and six cysteine residues, respectively (Figure 7A). These results also indicated that all cysteine residues in the lunasin complex are involved in disulfide bonds and that there are 2 disulfide bonds between lunasin and the 8975.1 Da subunit.

The mass of the 8975.1 Da subunit of the lunasin complex matched very well with the expected mass of the soybean 2S albumin large subunit (GenBank AAB71140.1). Analysis of peptides in a tryptic digest of the 8975.1 Da subunit by LC/MS/MS confirmed that this subunit does correspond to the 2S albumin large subunit (Table 1). Based on these results, it is clear that the lunasin complex represents the processed form of the pre-propeptide encoded by the soybean 2S albumin gene (GenBank: AF005030.1). The complete sequence of the 2S pre-propeptide and the relative positions of lunasin and the large subunit are shown in Figure 7B.

### Pilot-scale purification of lunasin

**Figure 8:**
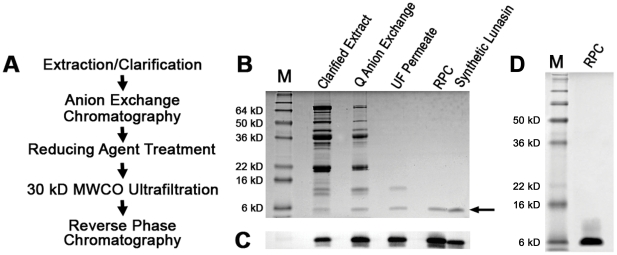
Pilot scale lunasin purification. A) Flow diagram of the optimized lunasin purification method. (B) Coomassie-stained SDS-PAGE gel of protein samples representing each stage of the pilot-scale purification. SDS-PAGE using a 15% Tris-glycine gel and diluted samples of clarified extract (1∶20), Q anion-exchange fraction (1∶40), UF permeate (1∶20), and RPC fraction (1∶40). Synthetic lunasin (500 ng) was loaded as a positive control. Molecular weight standards (M) are shown in the first lane. (C) Immunoblot analysis of protein samples representing each stage of pilot-scale purification. Proteins separated by SDS-PAGE as described for (B) were transferred to a PVDF membrane and probed with a lunasin-specific mouse monoclonal antibody. Lunasin was detected in all the samples as a band with an apparent molecular weight of ∼5 kDa. (D) Coomassie-stained SDS-PAGE gel of final RPC-purified lunasin product. SDS-PAGE was performed on a 15% gel using 10 µg of RPC-purified lunasin. Molecular weight standards (M) are shown in the first lane.

To test the scalability of our purification scheme and determine the amount of lunasin that can be obtained with these methods, we utilized the pilot-scale facility available at Kentucky BioProcessing (Owensboro, KY, USA). For this study we began with 20.8 kg of soybean white flake and followed the process outlined in Figure 8A. The separations obtained using larger volume Q-Sepharose FF and reversed-phase columns were very similar to that observed during our bench-scale purifications (Figure 8B and 8C). SDS-PAGE analysis of the final lunasin product indicated that the lunasin we obtained is >99% pure (Figure 8D). Based on quantification of the protein band intensities obtained from Coomassie-stained SDS-PAGE gels of protein samples taken at each step of the purification process, we obtained an overall yield of lunasin from the initial extract of 20%, with a total of 9.2 g of lunasin recovered. This successful pilot-scale purification demonstrates that our purification method is scalable and that commercial-scale production of kilogram quantities of highly purified lunasin is possible.

### Biological activity of purified lunasin

**Figure 9:**
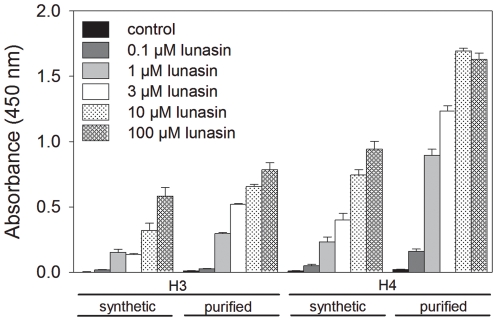
*In vitro* histone-binding of synthetic and purified lunasin to core histones H3 and H4. *In vitro* binding of synthetic and purified lunasin (0.1, 1, 10 and 100 µM) to human recombinant core histones H3 and H4 was assessed utilizing a modified ELISA format using 500 ng of either H3 or H4 as the capture protein. Both synthetic and purified lunasin bound to both histones H3 and H4 in a dose dependant manner. However, while both synthetic and purified lunasin bound to histone H3 with similar affinity, the purified lunasin exhibited a higher affinity for histone H4 when compared to its synthetic counterpart. Error bars represent +/− SD of 5 independent experiments were each sample was assayed in triplicate. Similar results were obtained when either 100 ng or 300 ng of H3 or H4 were used (data not shown).

Previous studies have demonstrated that a key component of the biological activity of lunasin is its ability to bind to the core histones H3 and H4 and modulate histone acetylation [23], [24]. To assess the potential biological activity of our purified lunasin, we performed a series of histone-binding experiments using a modified enzyme-linked immunosorbent assay (ELISA). For these experiments, synthetic lunasin and lunasin purified from white flake were compared for their ability to bind recombinant human core histones H3 and H4. These assays demonstrated that our purified lunasin binds to both histones H3 and H4 at levels equivalent to, and in some instances better than, synthetic lunasin (Figure 9). Both the synthetic and purified white flake-derived lunasins exhibited higher binding to histone H4 when compared to H3. Purified lunasin exhibited higher binding to histone H4 compared to that of the synthetic lunasin. These results demonstrate our purification method produces biologically active lunasin that is functionally equivalent to synthetic lunasin. These results also suggest that the C-terminal asparagine residue present in the purified soy-derived lunasin does not significantly affect lunasin's biological activity.

## Discussion

The recent advances in studies of the biological activities of lunasin and the potential use of lunasin as an anti-inflammatory or anticancer agent provide a strong rational for developing a robust and scalable purification method for lunasin that would generate large quantities of highly purified peptide suitable for large-scale animal and human trials. Although partially purified lunasin preparations may be adequate for oral administration as a nutraceutical, highly purified lunasin will be required to develop therapeutics. Previous studies have described methods that produce lunasin preparations that are ∼80–85% pure [20], [30]. These published methods utilize DEAE anion-exchange chromatography followed by size-exclusion chromatography using either Superdex 75 [30] or a 7 kDa resin [20]. The 75 kDa size-exclusion step did not provide good resolution between the protein components in the anion-exchange purified preparation, albeit, specific fractions were identified that appeared to be highly enriched for the ∼5 kDa lunasin peptide. This method yielded lunasin at a purity of ∼80%. Improved resolution was obtained with the 7 kDa resin which was associated with a modest increase in purity to 85%, albeit, the authors do not show a Coomassie-stained SDS-PAGE gel and indicate that purity was determined using Western blot analysis via an undescribed method. Although these purification schemes represent a good start, higher purity would be preferable to ensure that all the biological effects attributed to lunasin in these preparations are not related to contaminants. Moreover, size-exclusion chromatography is a more difficult method to scale since good separation requires that small sample volumes relative to the column volume must be applied.

We have developed an alternative method for purifying lunasin that utilizes an initial anion-exchange step followed by ultrafiltration using 30 kDa MWCO membranes and reversed-phase chromatography that represents a significant improvement over existing methods. This method yields highly purified lunasin (>99%) that is fully biologically active as assessed by histone binding. During the development of this method, we determined that the majority of the ∼5 kDa lunasin peptide in the initial white flake extract was found in a ∼14 kDa protein complex. This observation suggests that de Mejia and colleagues [20], [30] may have been purifying only the ∼20% of free lunasin present in white flake extracts that is not present in the ∼14 kDa protein complex. By adding a reduction step to the purification method to release lunasin from the ∼14 kDa protein complex, we significantly improved the yield of lunasin peptide.

We investigated the precise nature of the lunasin peptide obtained using our purification method by conducting an extensive MS analysis of the purified lunasin and the purified ∼14 kDa protein complex. Characterization of the purified lunasin by ESI-MS revealed that the major peptide in this preparation has a monoisotopic mass of 5139.25. This corresponds to the sequence SKWQHQQDSCRKQLQGVNLTPCEKHIMEKIQGRGDDDDDDDDDN and indicates that soy-derived lunasin has an asparagine residue at the C-terminus. LC-MS/MS analysis of tryptic peptides derived from purified lunasin confirmed the presence of the C-terminal asparagine. This is consistent with a previous analysis of soybean 2S albumin encoded by the AL3 gene [32]. It is not clear why two other previous studies did not detect the presence of the C-terminal asparagine when partially purified lunasin from soybean was analyzed using matrix-assisted laser desorption/ionization-time of flight (MALDI-TOF) analysis [20], [30]. The linear TOF method used by these researchers is less precise than the methods we used, and may not detect a 0.1 Da difference in peptide mass in some cases. However, in both of these publications the reported mass for purified lunasin was 5.1 kDa, which is consistent with our analysis. Moreover, Dia et al. [30] also presented data for synthetic lunasin with the sequence SKWQHQQDSCRKQLQGVNLTPCEKHIMEKIQGRGDDDDDDDDD and reported a mass of 5.0 kDa. Taken together, these previous reports are consistent with our results and indicate that the lunasin peptide from soybean contains 44 amino acids and has a C-terminal asparagine immediately following the aspartic acid tail. The presence of the C-terminal asparagine is consistent with the published cDNA sequence (GenBank: AF005030.1).

MS analysis of the purified ∼14 kDa complex and the gel-purified ∼9 kDa protein isolated from the complex after reduction with DTT demonstrated that the complex corresponds to the processed GM2S albumin protein. This is consistent with reports that lunasin copurifies with the large subunit of the GM2S protein [20], [30], [32]. Analysis of the complex after reduction and alkylation with IAA demonstrated that the complex contains four disulfide bonds, and that two of these disulfide bonds are between the two cysteines in lunasin and two of the six cysteines present in the GM2S large subunit. Two additional disulfide bonds were found to be present between the four remaining cysteines present in the GM2S large subunit. Taken together, these results support the conclusion that the soybean GM2S albumin protein is expressed and processed in a manner similar to that of other plant 2S albumins [32], [33], [34], [35].

To test the scalability of our lunasin purification method, we conducted a pilot scale production trial at Kentucky BioProcessing. This trial started with 20.8 kg of soybean white flake and utilized an up-scaled version of our optimized method. A total of 9.2 g of lunasin at a purity of >99% was recovered, representing a 20% yield from the initial amount of lunasin present in the white flake extract. This yield was very similar to the yields we obtained performing the purification method at the bench scale. Given the fact that the pilot facility at Kentucky BioProcessing replicates efficiently to their commercial-scale facility, it will be possible to further upscale this method to produce kilogram quantities of highly purified lunasin for use in the develop of new lunasin-based therapeutics for the treatment of cancer and inflammatory diseases.

## Materials and Methods

### Reagents

All chemicals were ACS grade or better and were purchased from Sigma-Aldrich (St. Louis, MO, USA) except sodium phosphate (dibasic anhydrous and monobasic monohydrate) were from EMD Chemicals (Gibbstown, NJ, USA) and sodium chloride, Tris-base, glycine, and bovine serum albumin (BSA) were from Fisher Scientific (Pittsburgh, PA, USA). Defatted soy flour (white flake) was prepared and provided by Owensboro Grain (Owensboro, KY, USA). Briefly, de-hulled soybeans were processed in a flaking roll and then further processed by conveying the flake through an expander to form a collet. The collet was transferred to a solvent extractor where the oil was removed by extensive washing with hexane. The defatted flake was then air-dried under fans at ambient temperature to remove the hexane. The white flake was stored at ambient temperature until used. Synthetic lunasin peptide along with a lunasin-specific mouse monoclonal lunasin antibody were from GenScript Corporation (Piscataway, NJ, USA). The lunasin-specific mouse monoclonal antibody was raised against the synthetic peptide CEKHIMEKIQGRGDD (98.7% pure) conjugated to keyhole limpet hemocyanin. Most of our studies were done using the lunasin-specific monoclonal antibody that was raised using the peptide CEKHIMEKIQGRGDD as the antigen. Initial studies were performed using a lunasin-specific rabbit polyclonal primary antibody and synthetic lunasin provided by Dr. Ben O. de Lumen (University of California-Berkeley, USA). Horse-radish peroxidase (HRP)-conjugated sheep anti-mouse and HRP-conjugated goat anti-rabbit antibodies were purchased from Jackson ImmunoResearch (West Grove, PA, USA). Human, recombinant histones were purchased from New England BioLabs (Ipswich, MA, USA). All chromatography columns and resins were obtained from GE Healthcare (Piscataway, NJ, USA). Ultrapure water was by generated using a Milli-Q Synthesis system (Millipore, Billerica, MA, USA).

### Protein and SDS-PAGE analyses

Protein concentrations were determined using a bicinchoninic acid-based assay (BCA Protein Assay Reagent, Thermo Scientific, Rockford, IL, USA). BSA was used as a standard for crude and partially purified lunasin samples whereas synthetic lunasin was used as a standard for the highly purified lunasin samples.

SDS-PAGE was performed using 15% PAGEr Gold Tris-Glycine PreCast gels (Lonza, Rockland, ME, USA) according to the manufacturer's recommendations. Molecular weight standards correspond to SeeBlue® Plus2pre-stained proteins (Invitrogen/Life Technologies, Grand Island, NY, USA). Gels were fixed in 40% ethanol/10% acetic acid, stained with Coomassie Brilliant Blue 250 (Fluka/Sigma-Aldrich, St. Louis, MO, USA), and destained with a 7% isopropanol/5% acetic acid solution. Gels were imaged using a Kodak Image Station 4000R Pro (Carestream, Rochester, NY, USA) or an ImageQuant-RT ECL (GE Healthcare, Piscataway, NJ, USA) and individual protein bands quantified using Carestream Molecular Imaging Software version 5.0.

### Immunoblot analysis and enzyme-linked immunosorbent assays (ELISA)

SDS-PAGE gels were run as previously described to perform immunoblot analysis. Proteins were transferred to Immobilon-P 0.45 um PVDF membranes (Millipore, Billerica, MA, USA) at 20 V for 90 min at 4°C. Five percent (w/v) instant non-fat dry milk in Tris-Tween buffered saline (TTBS; 16.1 mM Tris-HCl/3.88 mM Tris-base/150 mM NaCl/0.5% Tween 20, pH 7.5) was used as a blocking reagent. Two washes of TTBS were performed prior to incubation with primary antibody for 90 minutes. The lunasin mouse monoclonal primary antibody was used at a 1∶75,000 or 1∶100,000 dilution into primary antibody solution (TTBS/0.5% BSA/0.04% NaN_3_). The lunasin polyclonal rabbit primary antibody was used at a 1∶5000 dilution. Three washes with TTBS were performed prior to incubation with the appropriate secondary antibody for 60 minutes. A 1∶100,000 dilution of the HRP-conjugated sheep anti-mouse secondary antibody or HRP-conjugated goat anti-rabbit secondary antibody in 1% (w/v) instant non-fat dry milk in TTBS was used. Three washes with TTBS were performed before incubating with the chemiluminescent detection solution (ECL Advance™ Western Blotting Detection Kit, GE Healthcare, Piscataway, NJ, USA) and imaging using a Kodak Image Station 4000R Pro and Carestream Molecular Imaging Software version 5.0 (Carestream, Rochester, NY, USA). The image shown in Figure 2B was generated by first imaging the filter using white light to detect the pre-stained molecular weight standards, followed by imaging the filter after addition of the ECL Advance™ reagent and using the imaging software to merge the images to generate a composite image.

A direct ELISA was performed for quantitative measurements of lunasin concentration in partially-purified preparations. Samples were diluted into coating buffer (15 mM Na_2_CO_3_/35 mM NaHCO_3_/3 mM NaN_3_, pH 9.6), 50 µL aliquots of sample were added to wells of a 96-well plate (Nunc MaxiSorp™, Nalge Nunc International, Rochester, NY), and the plates were incubated for 60 minutes at 37°C. Wells were washed two times with PBST (137 mM NaCl, 2.7 mM KCl, 10.1 mM Na_2_HPO_4_, 1.8 mM KH_2_PO_4_, 0.05% Tween 20, pH 7.4) and then blocked with 150 µL per well of PBSTM 5% (PBST containing 5% w/v instant non-fat dry milk) for 60 minutes at room temperature or overnight at 4°C. The wells were then washed two times with ultrapure water. Lunasin primary antibody was prepared in PBSTM 1% (PBST containing 1% w/v instant non-fat dry milk) at a 1∶50,000 dilution. A 50 µL aliquot of diluted primary antibody was added to each well and incubated for 60 minutes at 37°C. The wells were then washed three times with ultrapure water. The HRP-conjugated secondary antibody was diluted to 1∶5000 in PBSTM 1% and 50 µL aliquots were added to each well prior to incubating for 60 minutes at 37°C. The wells were then washed three times with ultrapure water. The plate was developed using 50 µL per well of a tetramethyl benzidine-based reagent (TMBW; BioFX One Component HRP Microwell Substrate, SurModics, Eden Prairie, MN, USA) and an incubation time of four minutes at room temperature. The reaction was stopped with 50 µL per well of stop solution (0.6 N H_2_SO_4_/1 N HCl). The absorbance at 450 nm for each well was measured using a DTX 880 Multimode Detector (Beckman Coulter, Indianapolis, IN, USA). This ELISA format was useful for accurately measuring between 7 and 26 ng lunasin.

### Pilot-scale purification of lunasin

#### Extraction

For the large-scale purification of lunasin from soybean white flake, a 12.5∶1 extraction buffer (20 mM sodium phosphate/150 mM NaCl/20 mM ascorbic acid/10 mM sodium metabisulfite, pH 7.4) to white flake soy flour ratio was used. The white flake was suspended in extraction buffer and mixed for one hour. After mixing, a diatomite filter aid, Celpure 300 (Advanced Minerals Corporation, Santa Barbara, CA, USA), was added to the extract (33 g/L). The extract was passed through a filter press fitted with 1 micron M-503 filter pads (ErtelAlsop, Kingston, NY, USA) to produce a clarified extract. After filtering, the filter cake was blown dry with compressed air and a wash was performed using extraction buffer (wash volume: 50% of total extract). The wash was combined with the initial filtered extract to generate the final clarified extract.

#### Anion-exchange chromatography

All chromatography procedures were performed in clean room suites at Kentucky BioProcessing to ensure sterility of final product. Anion-exchange chromatography was performed using a 20.0×13.0 cm Q-Sepharose FF column on a Pharmacia 10 mm Bioprocess System Skid. The skid and column were both sanitized with 1 N NaOH and then pre-conditioned with 10 CV of equilibration buffer (Buffer A: 20 mM sodium phosphate/150 mM NaCl, pH 7.4) prior to applying samples. Clarified extract was applied onto the column through the sample inlet at a residence time between 2 and 2.77 minutes. The column was washed with 14.8 CV of equilibration buffer and the lunasin eluted using a linear gradient of NaCl in the elution buffer (Buffer B: 20 mM sodium phosphate/1 M NaCl, pH 7.4). Lunasin eluted from the column between 0.26 M and 0.50 M NaCl. The fractions containing lunasin were filtered through an inline 0.2 µm capsule filter and combined.

#### Reduction and ultrafiltration

The lunasin-containing fraction obtained by Q-Sepharose FF chromatography was brought to a final concentration of 2 mM DTT and stirred with an overhead mixer at room temperature for one hour. The DTT-treated fraction was subjected to ultrafiltration using five, 0.1 sq. meters each, 30 kDa MWCO polyethersulfone membranes using a Sartorius Sartocon Slice unit (Sartorius Stedium Biotech, Bohemia, NY, USA). Lunasin accumulates in the permeate fraction during this procedure. Ultrafiltration was continued until the retentate remaining in the sample reservoir reached a volume of ∼1 L. The retentate was then washed with five volumes of buffer (20 mM sodium phosphate/300 mM NaCl, pH 7.4) with each wash being reduced to a final volume of ∼1 L. Permeates generated from these washes were combined with the initial permeate for further purification.

#### Reversed-phase chromatography (RPC)

RPC was used as the final step in the purification process using a 10.0×9.2 cm Source 15RPC column on an AKTApilot™ system (GE Healthcare, Piscataway, NJ, USA). Prior to chromatography, the column was sanitized with 1 N NaOH and equilibrated with ten CV of equilibration buffer (20 mM sodium phosphate/150 mM NaCl, pH 7.4). The lunasin fraction was applied onto the column with a residence time of 2.5 minutes. A five CV wash with equilibration buffer was performed, followed by a step elution using 20%, 40%, 60%, 80%, and 100% elution buffer (Buffer B: 17 mM sodium phosphate/127.5 mM NaCl/15% n-propanol, pH 7.4). Fractions were collected at each gradient step; as expected, the 100% B gradient step was the lunasin-containing fraction.

Next, the lunasin-containing fraction obtained by RPC was concentrated using a 0.5 m^2^ 2 kDa cellulose cassette (Sartorius Stedium Biotech, Bohemia, NY, USA). Difiltration was performed to exchange the RPC elution buffer with 50 mM sodium phosphate, pH 7.4. The retentate and wash were collected and filtered through a 0.2 µm filter. The amount of lunasin present in the concentrated sample was determined using a BCA protein assay with synthetic lunasin as a standard. The lunasin concentrate was then diluted with 50 mM sodium phosphate, pH 7.4 to a final concentration of 4.65 mg/mL. Sterile, glass vials were each filled with 5.5 mL of final product and stored at 4°C.

### Mass spectrometry

#### Electrospray ionization mass spectrometry (ESI-MS) analysis of purified lunasin and lunasin complex

Purified lunasin complex was desalted with C_18_ ZipTip (Millipore, Billerica, MA) and ESI spectra of lunasin complex was obtained using an Orbitrap XL mass spectrometer (Thermo Scientific, San Jose, CA) equipped with TriVersa NanoMate system (Advion BioSciences, Ithaca, NY). The MS spectra were deconvoluted with Xtract (Thermo Scientific, San Jose, CA). To analyze subunits of lunasin complex, purified lunasin complex was reduced with 5 mM DTT at 70°C for 15 minutes, followed by alkylation with 15 mM iodoacetamide (IAA) at room temperature in the dark for 15 min. Reduced lunasin complex samples, with or without further alkylation, were desalted with C_18_ ZipTip and analyzed using an Orbitrap XL mass spectrometer.

#### LC-MS/MS Analysis of Lunasin Subunits

Gel-purified lunasin subunits were desalted with PepClean C_18_ spin column (Pierce/Thermo Scientific, Rockford, IL), reduced with DTT, alkylated with IAA, and incubated with sequencing grade modified trypsin (Promega, Madison, WI) at 37°C overnight. Incubation was stopped by adding 5% formic acid to the samples and the digests were loaded on to a Hypersil Gold C_18_ column and separated using an Accela HPLC system (Thermo Scientific, Waltham, MA, USA) with an aqueous acetonitrile/0.1% formic acid gradient. The eluted peptides were directed to an Orbitrap XL mass spectrometer and MS/MS spectra of the peptides were acquired in data dependent scan mode.

### Histone-binding assay

Aliquots of recombinant human histones H3 or H4 stock solutions where the histone concentration was determined by measuring the absorbance at 280 nm were plated in 96 well MaxiSorp™ plates in coating buffer (15 mM Na_2_CO_3_/35 mM NaHCO_3_/0.02% NaN_3_/1 mM freshly prepared DTT, pH 9.6) to generate final quantities of 100, 300 and 500 ng/well. DTT was added at the time of plating to allow for even distribution and prevention of oligomerization of the histones. Negative controls consisted of wells containing coating buffer alone with no histones. Plates were sealed with adhesive plastic and incubated for 60 minutes at 37°C. Plates were then washed twice with PBST and the remaining protein-binding sites blocked for 60 minutes at room temperature in PBST containing 5% non-fat dry milk. Following two washes with ultrapure water, either synthetic or purified lunasin diluted in PBSTM1% was added to the wells at final concentrations of 0.1, 1, 10, 100 µM. Plates were incubated at 37°C for 60 minutes, washed three times with PBST and incubated for another 60 minutes at 37°C with the primary monoclonal mouse anti-lunasin antibody diluted 1∶5000 in PBSTM1%. After three washes with ultrapure water, the secondary HRP-conjugated sheep anti-mouse antibody diluted 1∶5000 in PBSTM1% was added and the plates incubated at 37°C for 60 minutes. Plates were then washed three times with ultrapure water. Detection of lunasin bound to the histones was accomplished by the addition of the HRP substrate SureBlue™ (KPL, Gaithersburg, MD, USA) followed by six minutes incubation in the dark at room temperature. The reaction was terminated by the addition of an equal volume of 1 N HCl and the absorbance at 450 nm for each well was measured using a DTX 880 Multimode Detector (Beckman Coulter, Indianapolis, IN, USA).

## Supporting Information

Figure S1 **Optimization of white flake extraction conditions.** Soybean white flake (20 g) was extracted using the indicated extraction solutions and time. Extracts were filtered through four layers of cheesecloth and one layer of miracloth before centrifugation for ten minutes at 10,000× *g* at 4°C. Supernatants were collected and the protein content of the clarified extracts was determined using a bicinchoninic acid assay prior to SDS-PAGE analysis using 15% Tris-glycine gels. Molecular weight standards (MW Std) are shown in the first lane of each gel. Arrows indicate the position of the protein band corresponding to lunasin. (A) Effect of extraction time on the yield of lunasin. White flake was extracted with 75.5 mM sodium phosphate/68.4 mM NaCl, pH 7.4 for the indicated times up to an overnight (O/N) period. Aliquots containing ∼30 µg total protein were analyzed for each sample. (B) Effect of pH on the yield of lunasin. White flake was extracted for 60 minutes with different pH buffers: 20 mM sodium acetate buffer was used for pH 5 and 5.5; 50 mM sodium phosphate buffer was used for pH 6.0, 6.5, PBS was used for pH 7.0 and 7.4; and 50 mM Tris buffer was used for pH 8.0 and 8.5. Aliquots containing ∼20 µg total protein were analyzed for each sample. (C) Effect of buffer to white flake ratio on lunasin extraction efficiency. White flake was extracted with 50 mM sodium phosphate/150 mM NaCl, pH 7.4 using the indicated buffer to white flake ratios (v/w) for 60 minutes. Aliquots containing ∼40 µg total protein were analyzed for the 5∶1 sample and ∼25 µg total protein was analyzed for the 10∶1 and 12.5∶1 samples. (D) Comparison of the extraction efficiencies of buffer and water at different solution to white flake ratios (v/w). White flake was extracted with either ultrapure water adjusted to pH 7.4 or 75.5 mM sodium phosphate/68.4 mM NaCl, pH 7.4) using the indicated ratios of extraction solution to white flake. All extraction solutions also contained 20 mM ascorbic acid and 10 mM sodium metabisulfite. Aliquots containing ∼25 µg total protein were analyzed for each sample.
(TIF)

Figure S2 **Disruption of the lunasin-containing complex by treatment with reducing agents.** SDS-PAGE analysis was performed using 15% Tris-glycine gels. Each lane contains 15.5 µg of purified lunasin-containing complex without or with the indicated concentration of a reducing agent. All samples were treated with reducing agents in a reaction volume of 100 µL and incubated at room temperature for 1 hour prior to preparing samples for SDS-PAGE analysis. Molecular weight standards (M) are shown in the first lane. (A) Treatment with 0, 14.3, 71.5, and 143 mM beta-mercaptoethanol (BME). (B) Treatment with 0, 1.0, 5.0, and 10.0 mM tris(2-carboxyethyl)phosphine (TCEP). (C) Treatment with 0, 0.2, 1.0, and 2.0 mM dithiothreitol (DTT).
(TIF)
