# Supplementary figures and images for: Scalable Purification and Characterization of the Anticancer Lunasin Peptide from Soybean
Source: PLoS One. 2012 Apr 13;7(4):e35409. doi: 10.1371/journal.pone.0035409 (PMC3326064; doi:10.1371/journal.pone.0035409)

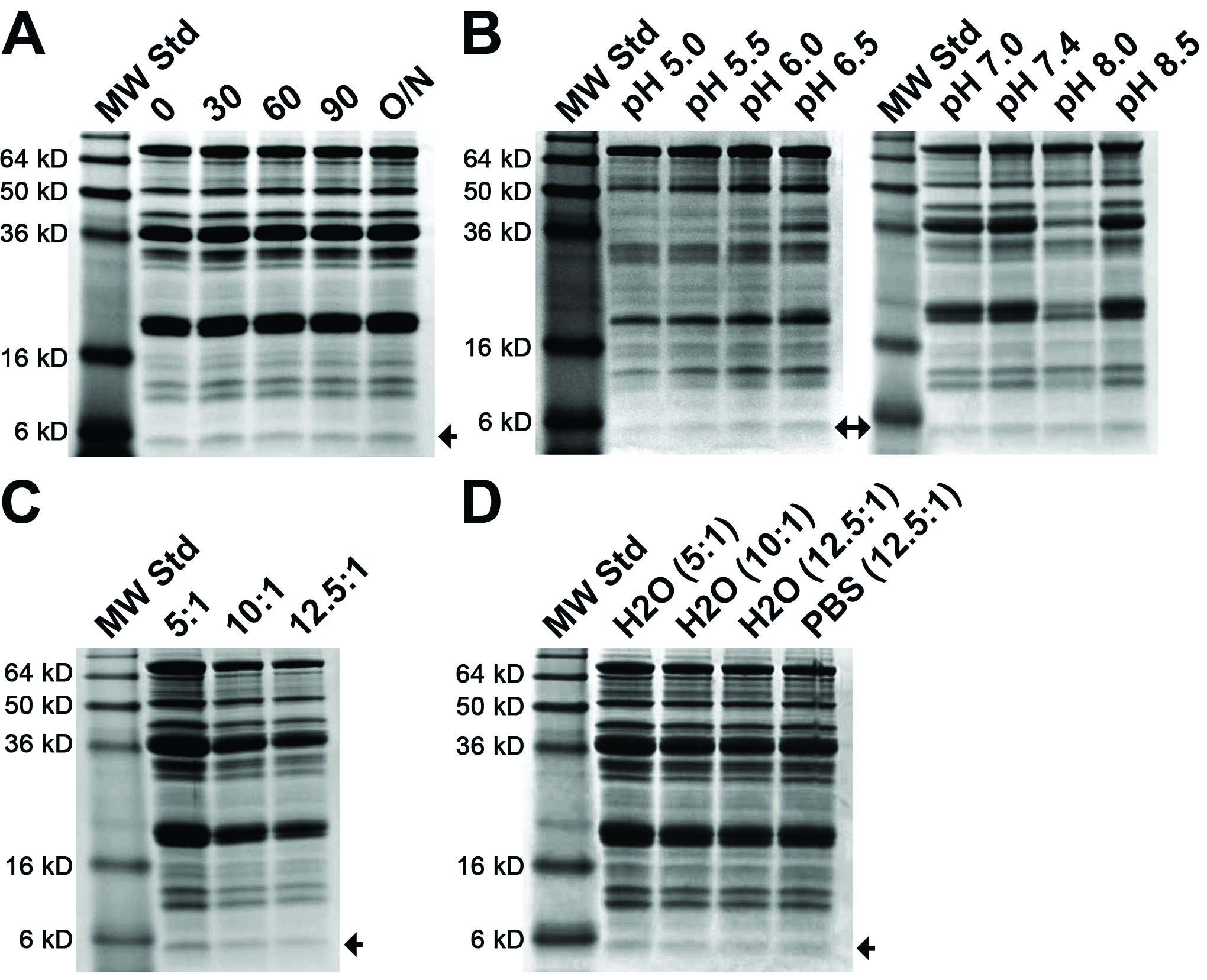

Supplement: Figure S1 — Optimization of white flake extraction conditions. Soybean white flake (20 g) was extracted using the indicated extraction solutions and time. Extracts were filtered through four layers of cheesecloth and one layer of miracloth before centrifugation for ten minutes at 10,000× g at 4°C. Supernatants were collected and the protein content of the clarified extracts was determined using a bicinchoninic acid assay prior to SDS-PAGE analysis using 15% Tris-glycine gels. Molecular weight standards (MW Std) are shown in the first lane of each gel. Arrows indicate the position of the protein band corresponding to lunasin. (A) Effect of extraction time on the yield of lunasin. White flake was extracted with 75.5 mM sodium phosphate/68.4 mM NaCl, pH 7.4 for the indicated times up to an overnight (O/N) period. Aliquots containing ∼30 µg total protein were analyzed for each sample. (B) Effect of pH on the yield of lunasin. White flake was extracted for 60 minutes with different pH buffers: 20 mM sodium acetate buffer was used for pH 5 and 5.5; 50 mM sodium phosphate buffer was used for pH 6.0, 6.5, PBS was used for pH 7.0 and 7.4; and 50 mM Tris buffer was used for pH 8.0 and 8.5. Aliquots containing ∼20 µg total protein were analyzed for each sample. (C) Effect of buffer to white flake ratio on lunasin extraction efficiency. White flake was extracted with 50 mM sodium phosphate/150 mM NaCl, pH 7.4 using the indicated buffer to white flake ratios (v/w) for 60 minutes. Aliquots containing ∼40 µg total protein were analyzed for the 5∶1 sample and ∼25 µg total protein was analyzed for the 10∶1 and 12.5∶1 samples. (D) Comparison of the extraction efficiencies of buffer and water at different solution to white flake ratios (v/w). White flake was extracted with either ultrapure water adjusted to pH 7.4 or 75.5 mM sodium phosphate/68.4 mM NaCl, pH 7.4) using the indicated ratios of extraction solution to white flake. All extraction solutions also contained 20 mM ascorbic acid and 10 mM [file pone.0035409.s001.tif]

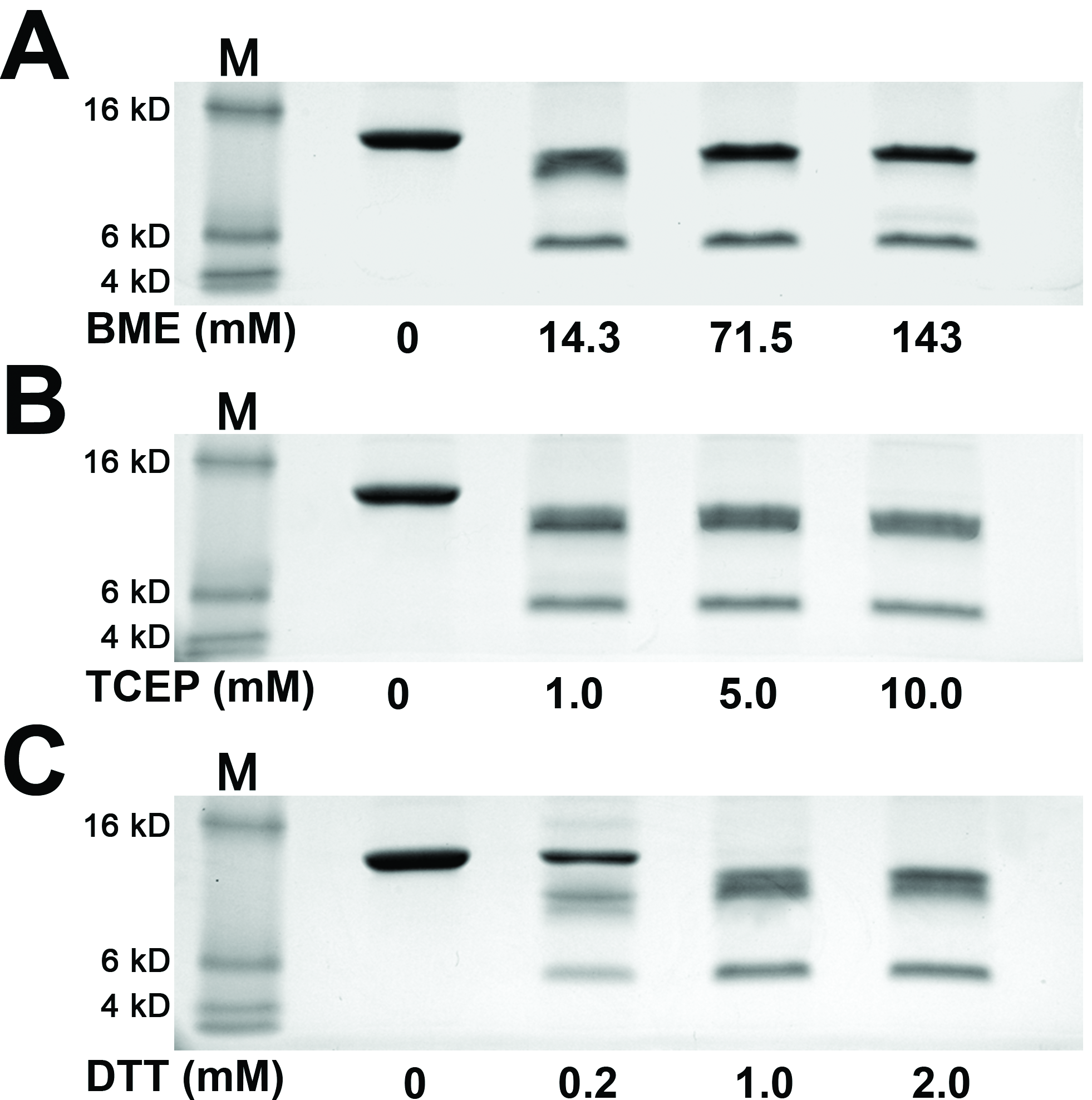

Supplement: Figure S2 — Disruption of the lunasin-containing complex by treatment with reducing agents. SDS-PAGE analysis was performed using 15% Tris-glycine gels. Each lane contains 15.5 µg of purified lunasin-containing complex without or with the indicated concentration of a reducing agent. All samples were treated with reducing agents in a reaction volume of 100 µL and incubated at room temperature for 1 hour prior to preparing samples for SDS-PAGE analysis. Molecular weight standards (M) are shown in the first lane. (A) Treatment with 0, 14.3, 71.5, and 143 mM beta-mercaptoethanol (BME). (B) Treatment with 0, 1.0, 5.0, and 10.0 mM tris(2-carboxyethyl)phosphine (TCEP). (C) Treatment with 0, 0.2, 1.0, and 2.0 mM dithiothreitol (DTT). (TIF) [file pone.0035409.s002.tif]
